# Interaction of 2′R-ochratoxin A with Serum Albumins: Binding Site, Effects of Site Markers, Thermodynamics, Species Differences of Albumin-binding, and Influence of Albumin on Its Toxicity in MDCK Cells

**DOI:** 10.3390/toxins10090353

**Published:** 2018-09-01

**Authors:** Zelma Faisal, Diána Derdák, Beáta Lemli, Sándor Kunsági-Máté, Mónika Bálint, Csaba Hetényi, Rita Csepregi, Tamás Kőszegi, Franziska Sueck, Benedikt Cramer, Hans-Ulrich Humpf, Miklós Poór

**Affiliations:** 1 Department of Pharmacology, Faculty of Pharmacy, University of Pécs, Szigeti út 12, H-7624 Pécs, Hungary; faisal.zelma@gytk.pte.hu; 2 János Szentágothai Research Center, University of Pécs, Ifjúság útja 20, H-7624 Pécs, Hungary; derdak.dia@gmail.com (D.D.); lemli.beata@gytk.pte.hu (B.L.); kunsagi-mate.sandor@gytk.pte.hu (S.K.-M.); ritacsepregi93@gmail.com (R.C.); koszegi.tamas@pte.hu (T.K.); 3 Department of General and Physical Chemistry, University of Pécs, Ifjúság útja 6, H-7624 Pécs, Hungary; 4 Department of Pharmaceutical Chemistry, Faculty of Pharmacy, University of Pécs, Rókus u. 2, H-7624 Pécs, Hungary; 5 Department of Pharmacology and Pharmacotherapy, Medical School, University of Pécs, Szigeti út 12, H-7624 Pécs, Hungary; monibalint18@gmail.com (M.B.); csabahete@yahoo.com (C.H.); 6 Department of Laboratory Medicine, Medical School, University of Pécs, Ifjúság útja 13, H-7624 Pécs, Hungary; 7 Institute of Food Chemistry, Westfälische Wilhelms-Universität Münster, Corrensstr. 45, 48149 Münster, Germany; f_suec01@uni-muenster.de (F.S.); cramerb@wwu.de (B.C.); humpf@wwu.de (H.-U.H.)

**Keywords:** 2′R-ochratoxin A, ochratoxin A, serum albumin, albumin-ligand interaction, species differences, cellular toxicity

## Abstract

Ochratoxin A (OTA) is a nephrotoxic mycotoxin. Roasting of OTA-contaminated coffee results in the formation of 2′R-ochratoxin A (2′R-OTA), which appears in the blood of coffee drinkers. Human serum albumin (HSA) binds 2′R-OTA (and OTA) with high affinity; therefore, albumin may influence the tissue uptake and elimination of ochratoxins. We aimed to investigate the binding site of 2′R-OTA (verses OTA) in HSA and the displacing effects of site markers to explore which molecules can interfere with its albumin-binding. Affinity of 2′R-OTA toward albumins from various species (human, bovine, porcine and rat) was tested to evaluate the interspecies differences regarding 2′R-OTA-albumin interaction. Thermodynamic studies were performed to give a deeper insight into the molecular background of the complex formation. Besides fluorescence spectroscopic and modeling studies, effects of HSA, and fetal bovine serum on the cytotoxicity of 2′R-OTA and OTA were tested in MDCK kidney cell line in order to demonstrate the influence of albumin-binding on the cellular uptake of ochratoxins. Site markers displaced more effectively 2′R-OTA than OTA from HSA. Fluorescence and binding constants of 2′R-OTA-albumin and OTA-albumin complexes showed different tendencies. Albumin significantly decreased the cytotoxicity of ochratoxins. 2′R-OTA, even at sub-toxic concentrations, increased the toxic action of OTA.

## 1. Introduction

**Figure 1:**
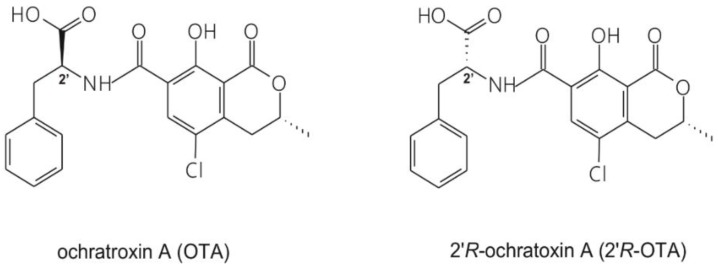
Chemical structures of the parent compound ochratoxin A (OTA) and its diastereomer 2′R-ochratoxin A (2′R-OTA).

Ochratoxin A (OTA; Figure 1) is a widespread mycotoxin produced by *Aspergillus* and *Penicillium* species [1]. It is present in several foods and drinks, mainly in cereals and cereal products but also in coffee, beer, wine, spices and cocoa [2,3]. Due to its wide occurrence and high thermal stability [4,5], eradication of OTA from the food chain seems impossible. OTA is nephrotoxic, however, other toxic effects (e.g., carcinogenic, teratogenic and immunotoxic) have also been attributed to it. Chronic exposure to OTA may play a role in the development of Balkan Endemic Nephropathy (BEN) [6,7]. During the roasting process of OTA-contaminated coffee, its diastereomer, 2′R-ochratoxin A (2′R-OTA, previously called 14-(*R*)-OTA; Figure 1) is formed (up to 26% of OTA can isomerize to 2′R-OTA), which has been detected in the blood of coffee drinkers [8]. Both mycotoxins appear in the circulation at the lower ng/L range, the concentration of 2′R-OTA may be even higher compared to OTA [9]. Based on in vitro studies, 2′R-OTA exhibits a lower acute toxicity than OTA [8,10].

Albumin is the most abundant plasma protein in the circulation, which maintains the oncotic pressure of blood and has buffering, antioxidant, as well as pseudo-enzymatic functions [11]. Albumin interacts with several endogenous and exogenous molecules and may significantly affect the pharmacokinetics/toxicokinetics of these compounds, due to modulation of their tissue distribution and elimination [11,12]. Sudlow’s Site I (in subdomain IIA) and Site II (in subdomain IIIA) are the major drug binding sites of human serum albumin (HSA) [11]. However, recent studies demonstrated the important role of the Heme binding site in subdomain IB [13]. Several mycotoxins form stable complexes with albumin, including aflatoxins, citrinin, ochratoxins, and zearalenone [14,15,16,17,18]. Interestingly, OTA forms a highly stable complex with HSA, leading to its extremely high plasma protein binding (99.8%), and long plasma elimination half-life (approximately one month) in humans [19,20]. The primary binding site of OTA is located in Sudlow’s Site I on HSA, where ionic interactions of the dianionic OTA (containing deprotonated carboxyl and phenolic hydroxyl groups) with two arginine moieties (R222 and R257) further stabilize the complex [21,22]. Furthermore, unusually large species differences in OTA-albumin interaction have been observed: The stability of OTA-HSA complex is approximately tenfold, and 30-fold higher respectively than the stabilities of OTA complexes formed with bovine and rat albumins [6,17]. 2′R-OTA binds to HSA with approximately a tenfold lower affinity than OTA [23]. Despite its lower affinity, the 2′R-OTA-HSA complex is very stable: The biding constant of this complex (*K* = 2 × 10^6^ L/mol) is tenfold higher compared to citrinin-HSA (*K* = 2 × 10^5^ L/mol) or zearalenone-HSA (*K* = 10^5^ L/mol) complexes [15,18,23], and 100-fold higher than that of aflatoxin-HSA complexes [14]. Similarly to OTA, 2′R-OTA occupies the Sudlow’s Site I as its high-affinity binding site on HSA, close to Trp-214, which allows energy transfer between HSA and the albumin-bound 2′R-OTA molecule [23]. Since 2′R-OTA also forms very stable complexes with albumin and albumin-binding strongly influences the toxicokinetics of OTA, the deeper understanding of 2′R-OTA-albumin complex formation is desired.

In this study, further aspects of 2′R-OTA-albumin interactions were examined (and compared with OTA-albumin complexation), including effects of site markers on the albumin-binding of 2′R-OTA, thermodynamics of 2′R-OTA-HSA complex formation, species differences of its albumin binding (human, bovine, porcine and rat albumins were tested), and influence of albumin on the acute cellular toxicity of 2′R-OTA, in the absence and presence of the parent compound OTA. Thermodynamic and modeling studies give a deeper insight into the physicochemical background of the complex formation. Displacement of 2′R-OTA from albumin may strongly influence the elimination half-life and/or tissue distribution of the mycotoxin. The effect of site markers on the albumin-binding of 2′R-OTA demonstrates which type of molecules are able to disrupt the albumin-binding of 2′R-OTA. The significant interspecies differences regarding the elimination half-life of OTA is mainly attributed to its different binding affinity toward albumins from various species [6,19]. Therefore, investigation of the complex formation of 2′R-OTA with albumins from different species is reasonable. It may also help to estimate how effectively we can extrapolate the results of animal studies to humans. Furthermore, we aimed to test in cell experiments the hypothesis that high binding constants of 2′R-OTA-albumin complexes result in the entrapment of 2′R-OTA in the cell medium, leading to the poor cellular uptake and the decreased in vitro toxicity of the mycotoxin. We demonstrate that albumin significantly alleviate the cytotoxicity of 2′R-OTA (and OTA) in MDCK cell line. Finally, we aimed to investigate the influence of 2′R-OTA on the cytotoxicity of OTA in the absence and presence of albumin. This study highlights, when simultaneously present 2′R-OTA with OTA, 2′R-OTA can increase the OTA-induced loss of cell viability despite its relatively lower toxicity.

## 2. Results and Discussion

### 2.1. Binding of 2′R-OTA and OTA to HSA Based on Modeling Studies

**Figure 2:**
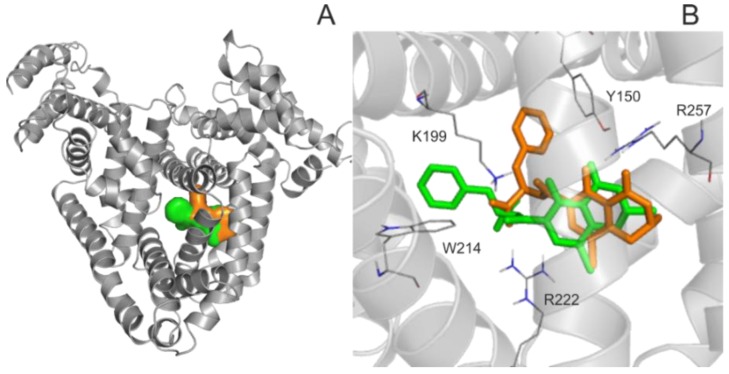
Binding site and position of 2′R-OTA verses OTA in Human serum albumin (HSA). (**A**) HSA is represented with grey cartoon, and ligand conformation bound to Sudlow’s Site I are orange (2′R-OTA^−^) and green (2′S-OTA^−^). (**B**) The binding differences observed between 2′R-OTA^−^ (orange) and 2′S-OTA^−^ (green). The ligands are represented with colored thin sticks, while the protein binding site with grey cartoon. Hydrogen atoms of the docked ligand conformations are not presented.

**Figure 3:**
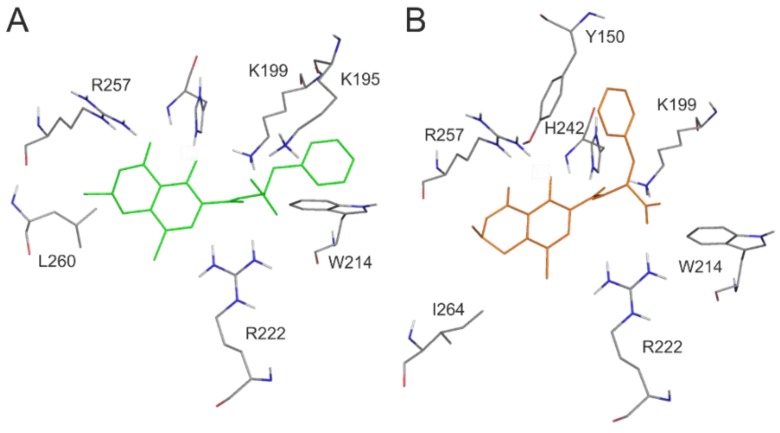
Interaction of monoanionic forms of OTA and 2′R-OTA with HSA. Amino acids of HSA (grey sticks) are presented with the docked conformation of 2′S-OTA^−^ (green sticks; **A**) and 2′R-OTA^−^ (orange sticks; **B**). Hydrogen atoms of the docked ligand conformations are not presented.

**Figure 4:**
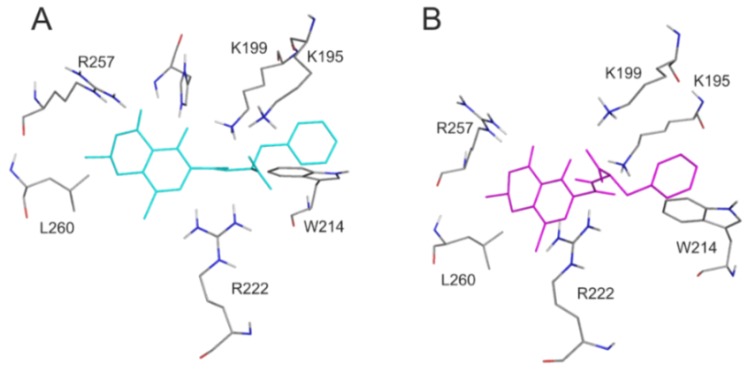
Interaction of dianionic forms of OTA and 2′R-OTA with HSA. Amino acids of HSA (grey sticks) are presented with the docked conformation of 2′S-OTA^2−^ (cyan sticks; **A**) and 2′R-OTA^2−^ (magenta sticks; **B**). Hydrogen atoms of the docked ligand conformations are not presented.

For the deeper insight into the binding position of 2′R-OTA in HSA (compared to OTA), modeling studies have been performed. As previous studies demonstrated, OTA is present in both monoanionic (OTA^−^) and dianionic (OTA^2−^) forms at physiological pH [24,25,26]. However, in the human circulation, OTA^-^ is rapidly deprotonated by HSA resulting in the formation of an ion pair by its dianionic form with two arginine moieties of HSA (R222 and R257) [21]. The deprotonation step implies structural modifications both on HSA and the ligand molecule. Since this mechanism is a dynamic process, such modifications cannot be reliably captured with static docking calculations. Using the apo structure of HSA, docking calculations with AutoDock 4 resulted in relevant structural information about the formation of OTA^−^-HSA complex (initial stage), while experimental analysis provides information regarding OTA^2−^-HSA complex (final stage). The location of OTA binding site on albumin has been proposed by both computational [23,27] and experimental studies [22]. Our docking calculations focused on the previously reported binding site (Figure 2A), to investigate the differences in the binding mode between 2′R- and 2′S-OTA diastereomers (Figure 2B). After docking calculations, multiple ranks were obtained, but only the best ranked ligand conformation was selected for further discussions. Based on these calculations, OTA^−^ has the same orientation and binding conformation as the previously observed binding mode [27]. The oxygen atoms of the carbonyl and phenolic hydroxyl groups orient towards R257, while the carboxylic group towards amino acid R222 (Figure 3A). Interaction with R222, as an important amino acid that stabilizes 2′R-OTA-HSA complex, has been described in a recent computational molecular dynamics study as well [23]. A strong π-stacking with W214 was also observed for monoanionic and dianionic OTA (Figure 3A and Figure 4A) and for dianionic 2′R-OTA (Figure 4B). During the complex formation of monoanionic 2′R-OTA with HSA, π-stacking interaction of 2′R-OTA^−^ with Y150 was noticed (Figure 3B). Therefore, π-stacking between the phenyl group and W214 is not possible due to the switch of the phenyl group towards Y150. Therefore, the carboxylic moiety of 2′R-OTA^−^ also changes orientation, resulting in a weaker hydrogen bond with R222 and the absence of the interaction with K195 (Figure 3B); the latter was proposed to be among the residues that play an important role in stabilizing the phenyl ring [22]. The above listed differences between initial binding conformations of 2′R- and 2′S-OTA may provide a structural basis of the differences in experimental log*K* values described [23].

### 2.2. Effects of Site Markers on 2′R-OTA-HSA and OTA-HSA Interactions

**Figure 5:**
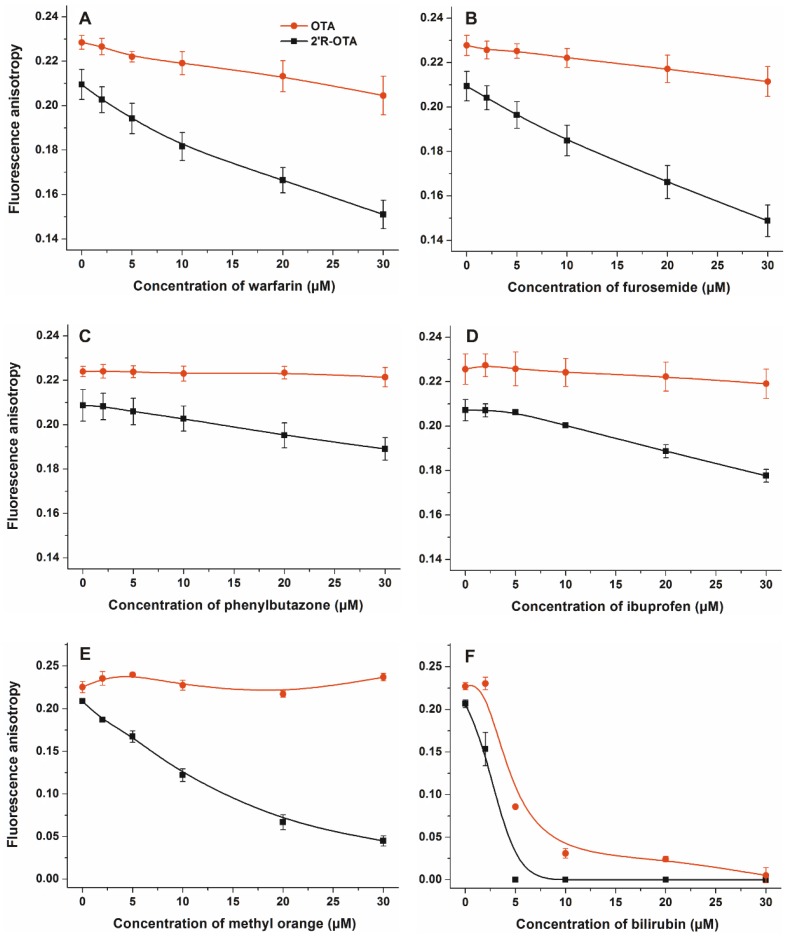
Fluorescence anisotropy values of mycotoxin-HSA complexes (1 µM OTA or 2′R-OTA and 1.5 µM HSA) in the presence of increasing concentrations of site markers (0–30 µM) in PBS (pH 7.4) ((**A)**:warfarin, (**B**) furosemide, (**C**) phenylbutazone, (**D**) ibuprofen, (**E**) methyl orange, and (**F**) bilirubin; λ_ex_ = 394 nm, λ_em_ = 447 nm).

Displacement of 2′R-OTA and OTA from HSA was investigated by fluorescence anisotropy in the presence of site markers. Site markers are ligand molecules occupying known and well-characterized binding sites in a protein (e.g., HSA). Therefore, site markers are commonly used compounds during the investigation of protein-ligand interactions. The displacement of a ligand molecule by a site marker demonstrates their competitive or allosteric interaction. Site markers can help to find the binding sites of ligand molecules and highlight which molecules are able to displace the test ligand from the protein. As it has been demonstrated, fluorescence polarization and anisotropy are suitable techniques to follow the complex formation of ochratoxins with albumin, including the displacement of ochratoxins from HSA by other compounds [15,23,24,28]. Site markers of Sudlow’s Site I (warfarin, furosemide and phenylbutazone), Sudlow’s Site II (ibuprofen) and Heme binding site (methyl orange and bilirubin) were applied to investigate their influence on the albumin-binding of ochratoxins [11,13]. In these experiments, anisotropy measurements were performed in PBS (pH 7.4), in the presence of ochratoxins and HSA (1.0 and 1.5 µM, respectively) with and without the site markers (0–30 µM; λ_ex_ = 394 nm, λ_em_ = 447 nm). As Figure 5 demonstrates, each site marker induced stronger decrease in the anisotropy value of 2′R-OTA compared to OTA. Fluorescence anisotropy gives information regarding the rotational freedom of fluorophores. The decreased anisotropy values of ochratoxins resulted from the increase of their rotational freedom, suggesting displacement of the mycotoxin from HSA [15,28]. These results support the previous observation that 2′R-OTA binds to HSA with much lower affinity than OTA [23]. Interestingly, each site marker induced some displacement of 2′R-OTA from HSA. Modeling studies and the energy transfer between Trp-214 residue of HSA and ochratoxins demonstrated that, similarly to OTA, 2′R-OTA occupies Sudlow’s Site I on HSA, as its high-affinity binding site [23]. However, the observation that other site markers displaced 2′R-OTA (and/or OTA) from albumin is not surprising, as these ligand molecules can allosterically modulate the complex formation. Site markers of Sudlow’s Site I (subdomain IIA) can competitively displace ochratoxins from albumin; however, because of the extremely high affinity of OTA towards HSA only slight displacement occurred (Figure 5A–C). Despite the lower stability of 2′R-OTA-HSA compared to that of the OTA-HSA complex, the binding constant of 2′R-OTA-HSA complex is very high (*K* ~ 2 × 10^6^ L/mol) [23]. Warfarin and furosemide produced strong displacement of 2′R-OTA from HSA (Figure 5A,B), whereas phenylbutazone induced a slight displacement (Figure 5C). Despite ibuprofen being one of the most commonly applied marker of Sudlow’s Site II (subdomain IIIA), we have demonstrated that high concentrations of ibuprofen can displace OTA from HSA [24]. Ibuprofen binds to HSA with comparable affinity to 2′R-OTA [12,23], however, it induced only slight displacement (Figure 5D), which also supports the allosteric nature of the interaction. Site markers of the Heme binding site (subdomain IB) induced the most significant displacement of 2′R-OTA from HSA (Figure 5E,F). This can be explained by the fact that the Heme binding site is allosterically coupled with the Sudlow’s Site I [11], therefore, it is reasonable to hypothesize that binding of methyl orange and bilirubin to subdomain IB induces structural changes on subdomain IIA, resulting in the significant decrease in the binding affinity of 2′R-OTA toward HSA. Furthermore, methyl orange binds with much lower affinity to HSA than bilirubin [13,29], which is also in agreement with our observation that bilirubin has stronger influence on the albumin-binding of 2′R-OTA. These results emphasize that, besides Sudlow’s Site I ligands, ligands of the Heme binding site can significantly interfere with the albumin-binding of ochratoxins, which has not been reported, to the best of our knowledge.

### 2.3. Thermodynamics of 2′R-OTA-HSA Interaction

**Figure 6:**
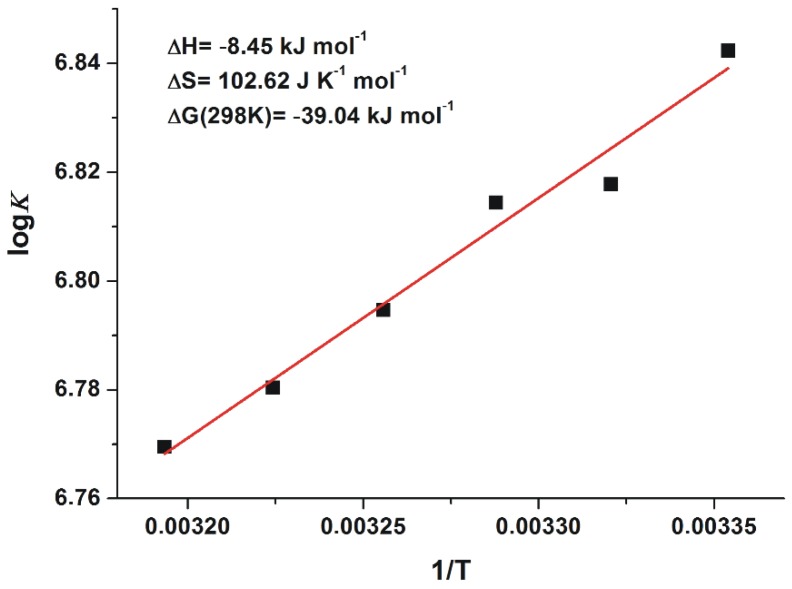
The van’t Hoff plot and thermodynamic parameters of 2′R-OTA-HSA complex formation.

Since thermodynamic data give a deeper insight into the nature of protein-ligand interactions (including binding forces are involved in the complex formation) [30], the binding ability of 2′R-OTA towards HSA was also examined at different temperatures. According to the van’t Hoff equation (Equation (2)), the log*K* values expressed as the function of the appropriate reciprocal temperatures show good linearity (Figure 6). The *ΔG* values suggest a non-covalent spontaneous interaction of 2′R-OTA with HSA at room-temperature (−39.04 kJ mol^−1^). Our results suggest that formation of 2′R-OTA-HSA complex is an entropy-driven process, where smaller enthalpy change is associated with higher entropy gain (in agreement with the known enthalpy-entropy compensation). Furthermore, based on the description of Ross and Subramanian [30], the negative enthalpy change with the positive entropy change suggest the importance of electrostatic forces in stabilization of the formed complexes.

### 2.4. Effects of Human, Bovine, Porcine and Rat Albumins on the Fluorescence Emission Spectra of 2′R-OTA and OTA

**Figure 7:**
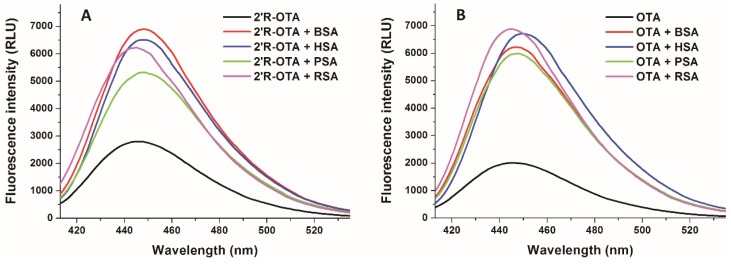
Fluorescence emission spectra of 2′R-OTA (**A**; 1 µM) and OTA (**B**; 1 µM) in the absence and presence of albumins from different species (each 5 µM) in PBS (λ_ex_ = 394 nm, λ_em_ = 447 nm; BSA, bovine serum albumin; HSA, human serum albumin; PSA, porcine serum albumin; RSA, rat serum albumin).

Since ochratoxins exhibit strong intrinsic fluorescence, molecular interactions of ochratoxins with albumin can be precisely followed by fluorescence spectroscopy [23,24]. Isomers can bind to the same protein with different affinity; therefore, it is reasonable to hypothesize that the fluorescence enhancement of 2′R-OTA and OTA by albumins may also show some differences. To test this hypothesis, the fluorescence emission spectra of 2′R-OTA (Figure 7A) and OTA (Figure 7B) were recorded in the absence and presence of albumins from different species, including human (HSA), bovine (BSA), porcine (PSA), and rat (RSA) serum albumins (λ_ex_ = 394 nm). Under the applied conditions, albumins gave negligible fluorescence emission signal at 447 nm. As Figure 7 demonstrates that each albumin caused significant increase in the fluorescence signal of ochratoxins, and slight changes in the emission wavelength maxima were observed as well. Interestingly, the fluorescence enhancement of ochratoxins by albumins from the other species showed different tendencies. The weakest increase in fluorescence was induced by PSA for both ochratoxins. However, 2′R-OTA showed the highest emission signal with BSA, whereas among the OTA-albumin complexes OTA-RSA complex expressed the highest fluorescence. Fluorescence enhancement (I/I_0_ values) of 2′R-OTA by albumins are demonstrated in Table 1.

### 2.5. Binding Constants of 2′R-OTA-albumin Complexes

**Figure 8:**
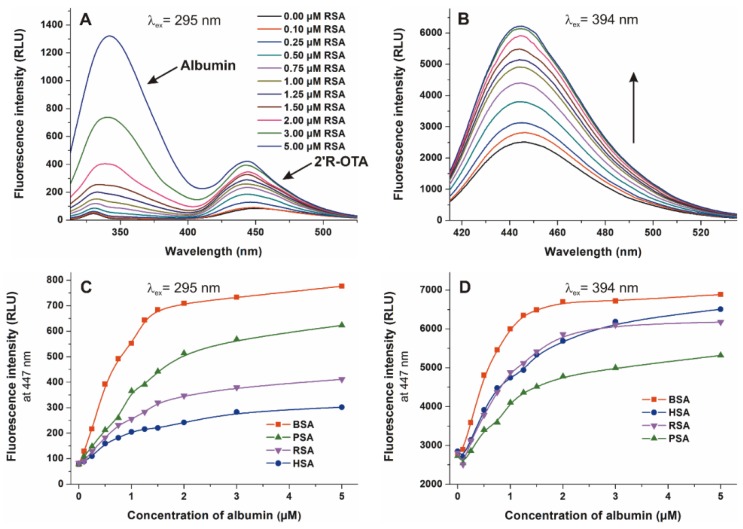
Fluorescence emission spectra of 2′R-OTA (1 µM) in presence of increasing concentrations of RSA (0–5 µM) in PBS (pH 7.4), using 295 nm ((**A**) ex. slit: 5 nm, em. slit: 5 nm) and 394 nm ((**B**) ex. slit: 5 nm, em. slit: 10 nm) excitation wavelengths. Fluorescence emission intensity of 2′R-OTA (1 µM) at 447 nm in the presence of increasing albumin concentrations (0–5 µM) using 295 nm (**C**) and 394 nm (**D**) excitation wavelengths.

Considering the previously published observations that the stability of OTA-albumin complexes show unusually large species differences [6,19], we aimed to evaluate the interaction of 2′R-OTA with human, bovine, porcine, and rat serum albumins. To determine the binding constants (*K*) of 2′R-OTA-albumin complexes, which demonstrates the stability of the formed complexes, the fluorescence emission spectra of 2′R-OTA (1 μM) were recorded in presence of increasing albumin concentrations (0–5 μM) in PBS (pH 7.4), using 295 nm (excitation of albumin) and 394 nm (excitation of 2′R-OTA) excitation wavelengths. Since the binding site of 2′R-OTA is located in Sudlow’s Site I (which is very close to the tryptophan amino acid in albumins), excitation of samples at 295 nm results in energy transfer between albumin and ochratoxins, and consequently the selective excitation of albumin-bound 2′R-OTA molecules [23,31]. However, excitation of the same samples at 394 nm leads to the excitation of both free and albumin-bound 2′R-OTA [23,24]. Figure 8 demonstrates the emission spectra and signals at both applied wavelengths. Using 295 nm as excitation wavelength, two emission peaks with their maxima approximately at 340 and 447 nm are observed. The first peak is the emission signal of albumin while the second peak is the fluorescence signal of albumin-bound 2′R-OTA (Figure 8A) [23]. Using 394 nm as excitation wavelength, the gradual increase in fluorescence at 447 nm can be observed in presence of albumins at increasing concentration (Figure 8B), due to the fluorescence enhancement of 2′R-OTA by albumins (Figure 7A). Binding constants of 2′R-OTA-albumin complexes were determined by the Hyperquad program, based on the fluorescence intensities measured at 447 nm (Figure 8C,D).

Table 2 demonstrates the decimal logarithmic values of the binding constants (*K*, unit: L/mol) of 2′R-OTA-albumin complexes. Despite OTA shows very large differences in binding affinity for albumins from different species [17,32], we did not observe such discrepancy of 2′R-OTA-albumin complexes. Both evaluations (using 295 nm or 394 nm as excitation wavelengths) suggest that 2′R-OTA binds to BSA and RSA with approximately 2–3 times higher affinity than to HSA and PSA, unlike OTA which showed the following affinity order in previous investigations: Human > bovine > porcine > rat [17,32]. Experiments using two excitation wavelengths to generate the emission spectra yielded some differences in the binding constants (Table 2). Using 295 nm as excitation wavelength, a slight spectral shift occurred in the peak associated with the 2′R-OTA molecule (Figure 8A,B). Therefore, it is reasonable to hypothesize that during the energy transfer from albumins to 2′R-OTA, a different orbital of 2′R-OTA is excited than during the direct excitation of the mycotoxin at 394 nm. As a result, a weaker enhancement of emission intensity was obtained, providing a slightly weaker complex stability.

### 2.6. Effects of Albumin on the In Vitro Toxicity of 2′R-OTA and OTA in MDCK Cell Line

**Figure 9:**
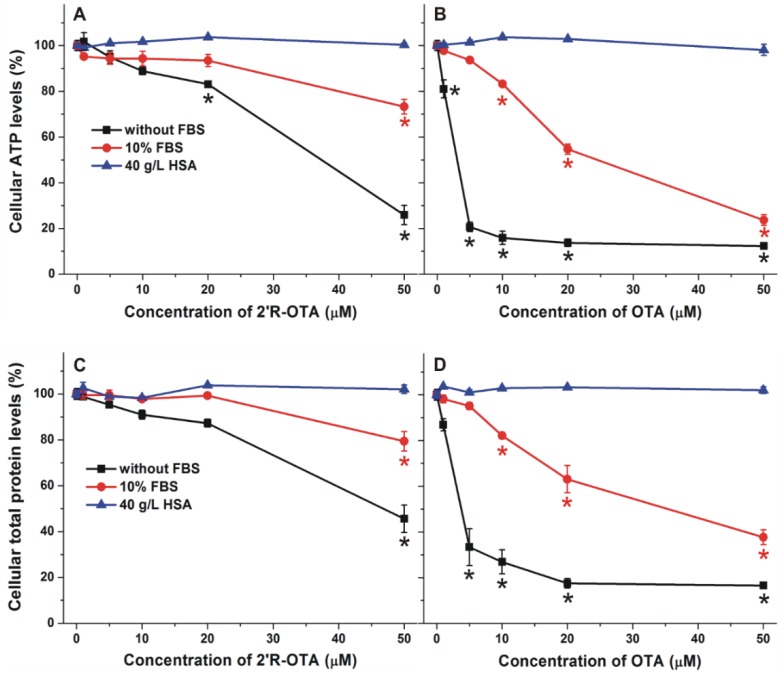
Effects of 2′R-OTA (**left**) and OTA (**right**) on cellular ATP (**top**) and total protein (**bottom**) levels of MDCK cells (% of control) in the absence and presence of 10% fetal bovine serum (FBS) or 40 g/L HSA, after 24 h incubation (* *p* < 0.01).

Similarly to OTA, 2′R-OTA also forms very stable complexes with albumins (Table 2). Therefore, it was reasonable to hypothesize that the presence of albumin may decrease the 2′R-OTA-induced toxicity in cell experiments, due to the entrapment of the mycotoxin in the cell medium by albumin. To test the influence of albumin on the in vitro effects of ochratoxins, the acute cellular toxicity of 2′R-OTA and OTA was investigated in the absence and presence of HSA and fetal bovine serum (FBS). MDCK kidney cells were treated with the mycotoxins at increasing concentrations (0–50 µM) for 24 h with and without 10% FBS or 40 g/L HSA. Ten percent FBS is commonly used in cell culture media (it yields approximately 3.5 g/L final concentration of BSA) in cell experiments, whereas approximately 40 g/L HSA circulates in the human blood. The mycotoxin-induced loss of cell viability was evaluated based on ATP and total protein levels. As Figure 9 demonstrates, 2′R-OTA and OTA caused dose-dependent, significant decreases in the concentration of ATP and total protein with slightly stronger effect on ATP levels, due to the ATP depleting effects of ochratoxins [33]. Under the applied conditions, the presence of 10% FBS or 40 g/L HSA (without ochratoxins) had no significant effect on cell viability (data not shown). In agreement with previous studies performed with IHKE (immortalized, human kidney, epithelial) and HepG2 (hepatocellular carcinoma, human liver, epithelial) cell lines [8,10], 2′R-OTA exhibited approximately 10 times lower cytotoxicity than OTA. Furthermore, the presence of 10% FBS significantly decreased, while 40 g/L HSA completely abolished the acute toxic action of ochratoxins on MDCK cells, which is in agreement with the report of Gekle et al. [34]. The weaker effect of FBS can be attributed to the lower BSA content of the cell medium (3.5 g/L BSA verses 40 g/L HSA). Since albumins form very stable complexes with both 2′R-OTA and OTA, albumin likely reduces the cellular uptake of these mycotoxins, resulting in their lower cellular toxicity.

### 2.7. Co-Treatment of MDCK Cells with OTA and 2′R-OTA in the Absence and Presence of FBS

**Figure 10:**
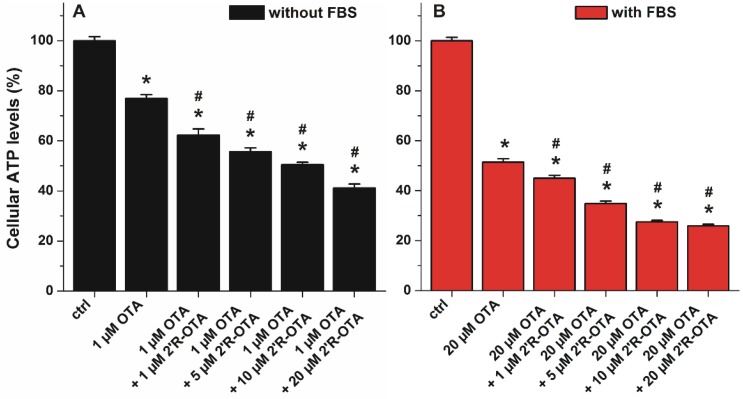
Effects of OTA with and without 2′R-OTA on MDCK cells in the absence (**A**) and presence (**B**) of 10% FBS, after 24 h incubation (compared to the control: * *p* < 0.01; compared to OTA-exposed cells: # *p* < 0.01).

Since 2′R-OTA appears in the circulation together with OTA, the toxic action of OTA in the presence of increasing 2′R-OTA concentrations was also tested. Both mycotoxins occupy Site I as their primary binding sites in albumin, therefore, 2′R-OTA may able to displace OTA from HSA, resulting in the increased cellular uptake and cytotoxicity of OTA in the presence of 2′R-OTA. Because 40 g/L HSA completely abolished the acute toxic action of ochratoxins, co-treatment of MDCK cells with OTA and 2′R-OTA was investigated without albumin and in the presence of 10% FBS. ATP levels/well were quantified after 24 h incubation. As ochratoxins were significantly less toxic in the presence of albumin, lower OTA concentration (1 μM) was applied in the experiments performed without FBS compared to the studies carried out with 10% of FBS (where 20 μM OTA was applied). As Figure 10 demonstrates, 2′R-OTA even at sub-toxic concentrations increased significantly the toxic effect of OTA both in the absence and presence of FBS. In the presence of FBS (Figure 10B), the 2′R-OTA-induced further loss in cell viability may be partly attributed to the displacement of OTA from albumin by 2′R-OTA. However, the co-treatment of MDCK cells with 2′R-OTA increases the toxic effect of OTA even in the absence of FBS (Figure 10A), suggesting that the displacement of OTA from albumin does not play a key role in the interaction. Therefore, other toxicokinetic and/or toxicodynamic mechanism(s) may be involved. Despite its relatively lower toxicity, when simultaneously present 2′R-OTA with OTA, 2′R-OTA can increase the effect of OTA.

## 3. Conclusions

In summary, the further aspects of 2′R-OTA-albumin interaction were examined and compared with OTA-albumin complex formation. The initial binding conformations of 2′R- and 2′S-OTA may provide a structural basis of the differences in experimental log*K* values. Since π-stacking between the phenyl group of 2′R-OTA and W214 in albumin is not possible, the carboxylic moiety of 2′R-OTA changes its orientation, leading to a weaker hydrogen bond with R222 and the absence of the interaction with K195. Site markers induced stronger displacement of 2′R-OTA than OTA from HSA, confirming further the lower binding affinity of 2′R-OTA toward HSA. Interestingly, not only Site I markers but the ligands of the Heme binding site (methyl orange and bilirubin) also strongly displaced 2′R-OTA from HSA. Based on thermodynamic studies, the formation of 2′R-OTA-HSA complex is an entropy-driven process, where electrostatic forces play an important role in the stabilization of the 2′R-OTA-albumin complexes. The fluorescence enhancement of 2′R-OTA and OTA by albumins from different species (human, bovine, porcine and rat) showed different tendencies: 2′R-OTA showed the highest emission signal with BSA while OTA exhibited the highest fluorescence with RSA. Despite the significant interspecies differences in the albumin-binding of OTA (human > bovine > porcine > rat) [17,32], the stability of 2′R-OTA-albumin complexes is similar. Similarly to OTA [34], the complex formation of 2′R-OTA with albumins alleviates or even abolishes the acute cellular toxicity of the mycotoxin, likely due to the decreased toxin uptake of MDCK cells. Despite its relatively low toxicity, even sub-toxic concentrations of 2′R-OTA can increase significantly the OTA-induced loss of cell viability, both in the absence and presence of fetal bovine serum. Therefore, these results also underline the high importance of mycotoxin co-exposure. Finally, we need to mention some limitations of our in vitro results. As it was concluded previously, albumin-binding of 2′R-OTA alone does not explain its longer plasma elimination half-life compared to OTA [23]. Furthermore, the toxicological consequence of OTA-albumin interaction is a controversial issue. The significant albumin-binding may limit the cellular uptake of OTA (even in vivo), which can be a protective mechanism. However, the complex formation of OTA with albumin also inhibits the rapid glomerular filtration of OTA (which would be an efficient elimination mechanism in the absence of albumin) [6]. Therefore, further in vivo experiments are necessary in the future to explore the toxicological outcome of 2′R-OTA-albumin and OTA-albumin interactions.

## 4. Materials and Methods 

### 4.1. Reagents

2′R-ochratoxin A (2′R-OTA) was synthetized as described [23]. Ochratoxin A (OTA), human serum albumin (HSA), bovine serum albumin (BSA), porcine serum albumin (PSA), rat serum albumin (RSA), racemic warfarin, furosemide, phenylbutazone, ibuprofen, methyl orange, bilirubin, and Dulbecco’s Modified Eagle Medium (DMEM) were obtained from Sigma-Aldrich (Saint Louis, MO, USA). Fetal bovine serum (FBS), (Pan-Biotech, Aidenbach, Germany), Bioluminescent ATP Assay Kit CLSII (Roche; Basel, Switzerland), and Coomassie Brilliant Blue G-250 (Reanal; Budapest, Hungary) were used as received. Stock solutions of 2′R-OTA and OTA (both 5000 µM) were prepared in ethanol (96 *v*/*v*%, Reanal, spectroscopic grade) and stored protected from light at −20 °C.

### 4.2. Modeling Studies

Mycotoxin molecules were built in Maestro [35]. The raw structures were energy minimized, employing the semi-empirical quantum chemistry program package, MOPAC [36], and PM6 parameterization [37]. The gradient norm was set to 0.001. The energy minimized structure was subjected to force calculations. The force constant matrices were positive definite. The minimized ligand structures were inputs of our docking calculations.

During the calculations, an apo crystallographic structure of HSA (PDB code: 1ao6) was used. Schrödinger Maestro program package v. 9.6 [35] was applied for the attachment of acetyl and amide capping groups to the N- and C-termini, respectively. As 1ao6 contains a homodimer structure, only chain A was used for calculations. Co-crystallized ions and water molecules were removed before minimizing the protein structure. A two-step protocol was applied using GROMACS software package [38], including a steepest descent and a conjugate gradient step, and using AMBER99-ildn force field [39]. Exit tolerance levels were set to 1000 and 10 kJ mol^−1^ nm^−1^ while maximum step sizes were set to 0.5 and 0.05 nm, respectively. The minimized target structure was the input of our docking calculations.

Using the optimized ligand and target structures, docking calculations were carried out with AutoDock 4.2 program [40] as described elsewhere [15,41,42,43]. Gasteiger-Marsilli partial charges were added to both the ligand and target atoms, using AutoDock Tools [40] and a united atom representation was applied for non-polar hydrogens. A grid box of 90 × 90 × 90 points, and 0.375 Å spacing was calculated and centered on Sudlow’s Site I pocket in AutoGrid 4. Lamarckian genetic algorithm was applied for global search. Flexibility was allowed on the ligand at all active torsions, number of docking runs was set to 100, numbers of energy evaluations and generations were 20 million [42]. Docked ligand conformations were ordered by the corresponding calculated interaction energy values and subsequently clustered using a tolerance of 1.75 Å root mean square deviation (RMSD) between cluster members [41]. Conformations of the lowest energy of a cluster were selected as cluster representatives were further analyzed.

### 4.3. Fluorescence Spectroscopic Studies

Fluorescence spectroscopic analyses, including anisotropy measurements, were carried out employing a Hitachi F-4500 fluorescence spectrophotometer. Spectroscopic measurements were performed in phosphate buffered saline (PBS, pH 7.4) at 25 °C in the presence of air. Fluorescence emission spectrum of 2′R-OTA (1 µM) was recorded in the presence of increasing concentrations of albumin (0.0, 0.1, 0.25, 0.5, 0.75, 1.0, 1.25, 1.5, 2.0, 3.0 and 5.0 µM) using 295 and 394 nm as excitation wavelengths. At 394 nm, we excited directly 2′R-OTA and its albumin complex [23]. However, using 295 nm excitation wavelength, albumin-bound 2′R-OTA was excited as a result of the energy transfer between albumins and 2′R-OTA [23,31].

Binding constants (*K*) of mycotoxin-albumin complexes were calculated by non-linear fitting, using the Hyperquad2006 program package (Protonic Software) as described in previous studies [23,44,45,46]. The stoichiometry of 2′R-OTA-albumin complexes was calculated based on the model associated with the lowest standard deviation.

Fluorescence anisotropy measurements were performed using 394 and 447 nm excitation and emission wavelengths, respectively. In these experiments, increasing amounts of site markers (final concentrations: 0, 2, 5, 10, 20, and 30 µM) were added to standard mycotoxin (2′R-OTA or OTA) and HSA concentrations (1.0 and 1.5 µM, respectively) in PBS. Fluorescence anisotropy were calculated using the following Equation:

(1) $$\;r=\frac{\left(I_{VV\;}-G\times I_{VH}\right)}{\left(I_{VV}+2\times G\times I_{VH}\right)}$$

where *r* and *G* denote the fluorescence anisotropy and the instrumental correction factor, respectively. *I_VV_* and *I_VH_* represent fluorescence emission intensities determined in vertical position of polarizer at pre-sample site and at vertical and horizontal position of post-sample polarizer, respectively.

### 4.4. Thermodynamic Studies

For the deeper insight into 2′R-OTA-HSA interaction, binding constants of mycotoxin-albumin complexes were also determined at six different temperatures (298, 301, 304, 307, 310 and 313 K). The binding constants were determined by the Hyperquad2006 program package [23,44,45,46], using 394 and 447 nm as excitation and emission wavelengths, respectively. Thermodynamic parameters associated to the complex formation between 2′R-OTA and HSA were determined based on the van ’t Hoff equation:

(2) $$\;logK=-\frac{\Delta G\;}{RT}=-\frac{\Delta H}{2.303RT}+\frac{\Delta S}{2.303R}$$

where *ΔG*, *ΔH*, and *ΔS* denote the Gibbs free energy, enthalpy, and entropy changes of the binding reaction, respectively; while *R* is the gas constant and *T* refers the temperature.

### 4.5. Cell Culturing and Cell Viability Assay

MDCK (Madin-Darby canine kidney epithelial cell line, ATCC: CCL-34) cell culture was maintained in DMEM with 10% FBS, penicillin (100 U/mL), and streptomycin (100 µg/mL) at 37 °C in a humidified environment (5% CO_2_). Cells were trypsinized and plated in 96-well sterile plastic plates (approximately 10^4^ cells/well) for overnight pre-incubation. Next day, the culture medium was replaced with fresh one (with or without 10% FBS or 40 g/L HSA), then cells were treated with increasing concentrations of 2′R-OTA and/or OTA (0, 1, 5, 10, 20 and 50 µM; in 150 µL medium/well). After 24 h incubation, cell viability was evaluated based on cellular ATP and total protein levels. Analyses of ATP and total protein concentrations/well were performed based on the luciferin-luciferase and Bradford reactions, respectively, during which the previously published method was applied without modifications [47].

### 4.6. Statistics

Figures demonstrate the means and standard error (SEM) values based on at least three independent experiments. Data were analyzed using one-way ANOVA test with *p* < 0.01, as the level of significance (IBM SPSS Statistics software 2012, version 21, New York, NY, USA).

## Figures and Tables

**Table 1:** Enhancement of the fluorescence of 2′R-OTA by complex formation with albumins from different species (human, bovine, porcine and rat).

Mycotoxin-Albumin Complex	I/I_0_ (±SD) (λ_ex_ = 394 nm, λ_em_ = 447 nm)
2′R-OTA-HSA	2.35 ± 0.07
2′R-OTA-BSA	2.48 ± 0.06
2′R-OTA-PSA	1.98 ± 0.05
2′R-OTA-RSA	2.22 ± 0.09

**Table 2:** Decimal logarithmic values of binding constants (*K*, unit: L/mol) of 2′R-OTA-albumin complexes. Log*K* values were determined based on emission spectra of 2′R-OTA, recorded at 295 and 394 nm as excitation wavelengths.

Mycotoxin-Albumin Complex	log*K* (± SD) (λ_ex_ = 295 nm, λ_em_ = 447 nm)	log*K* (±SD) (λ_ex_ = 394 nm, λ_em_ = 447 nm)
2′R-OTA-HSA	6.28 ± 0.05	6.64 ± 0.10
2′R-OTA-BSA	6.67 ± 0.07	7.32 ± 0.06
2′R-OTA-PSA	6.21 ± 0.09	6.84 ± 0.12
2′R-OTA-RSA	6.45 ± 0.03	7.19 ± 0.21
